# Distal Myopathies and Beyond: An Updated Overview of the Welander Distal Myopathy

**DOI:** 10.3390/cells15141302

**Published:** 2026-07-21

**Authors:** Ana García-Rubio, Julia Sánchez De los Santos, José M. Izquierdo

**Affiliations:** Centro de Biología Molecular Severo Ochoa, Consejo Superior de Investigaciones Científicas, Universidad Autónoma de Madrid (CSIC/UAM), Campus de Cantoblanco, C/Nicolás Cabrera 1, 28049 Madrid, Spain; anagar@ucm.es (A.G.-R.);

**Keywords:** distal myopathy, TIA1, Welander distal myopathy, pathophysiology

## Abstract

Myopathies are a heterogeneous group of disorders that primarily affect skeletal muscles and are classified as rare diseases owing to their low incidence. In particular, Welander distal myopathy is a rare, late-onset muscular disorder that was first described in 1951. Since then, substantial progress has been made in characterizing its clinical presentation and histopathological features. Despite these advances, many aspects of the disease, such as the underlying molecular mechanisms and factors driving phenotypic variability, remain unknown. Furthermore, the scarcity of reliable disease models not only limits the exploration of the molecular basis of the disease but also the development of effective treatments. In this review, we summarize the current knowledge of Welander distal myopathy and discuss potential directions in this field.

## 1. Introduction

Myopathies are a heterogeneous group of disorders characterized by alteration in skeletal muscle structure, metabolism, and channel function. These factors collectively contribute to an abnormal development and structural defects in sarcomeres, the fundamental units of muscle fibers [1,2]. Frequent symptoms of myopathies are stiffness, cramps, spasms, and muscle weakness which commonly compromises the performance of daily activities [1].

**Figure 1:**
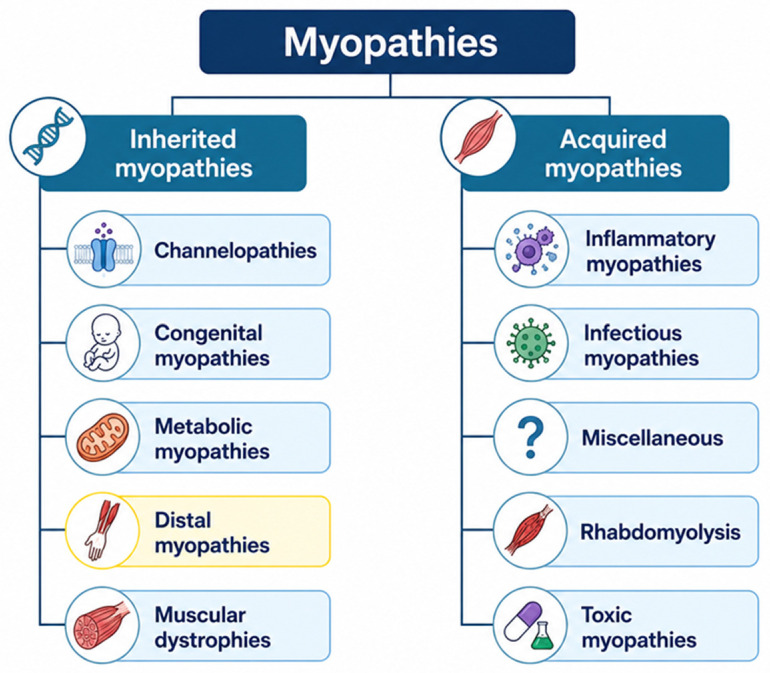
Classification of myopathies. Inherited myopathies encompass channelopathies, congenital myopathies, metabolic myopathies, muscular dystrophies, and mitochondrial myopathies, and—highlighted here—distal myopathies. Acquired myopathies include inflammatory, infectious, metabolic, toxic, and other miscellaneous forms. This schematic emphasizes the position of distal muscular dystrophies since it is the topic of this review. This figure was modified using ChatGPT Free (https://chatgpt.com/de-DE/plans/free/, accessed on 16 July 2026).

Myopathies may arise from inherited genetic disorders or metabolic defects, exposure to certain drugs and toxins, bacterial or viral infections, inflammatory processes, electrolyte disturbances, and hormonal irregularities [1]. Accordingly, based on their pathomechanisms and genetic backgrounds, myopathies are conventionally classified into two principal categories: inherited myopathies and acquired myopathies [2] (Figure 1).

Included in inherited myopathies, distal myopathies represent a distinct subgroup characterized by progressive weakness predominantly affecting distal muscles of the upper and lower limbs [3,4]. These disorders are genetically heterogeneous, and their subtypes differ in several features, including age at onset, distribution of muscle weakness, disease progression, and incidence [4].

Initially, before the causative gene was identified, the first distal myopathies were classified according to their clinical descriptions [5] resulting in the recognition of four principal distal myopathies: Welander distal myopathy (WDM), Miyoshi myopathy (MM), Nonaka distal myopathy (NDM) or distal myopathy with rimmed vacuoles (DMRV), and Udd distal myopathy (UDM) or tibial muscular dystrophy (TMD) [6,7,8]. This classification included several characteristics such as age of onset, initial muscle group involved or progression of the disease. Heritance was also studied and eventually the causative genes were identified [5].

Currently, apart from identifying the genes involved in the four distal myopathies mentioned previously, advances in molecular genetics have expanded the list of distal myopathies to more than 20 [3,7]. Mutations in distal myopathies are commonly found in genes of RNA-associated proteins (e.g., T-cell intracellular antigen 1 (TIA1)), sarcomere proteins (e.g., myosin heavy chain 7 (MYH7)) and membrane-associated proteins (e.g., caveolin 3 (CAV3)) [3,4].

**Figure 2:**
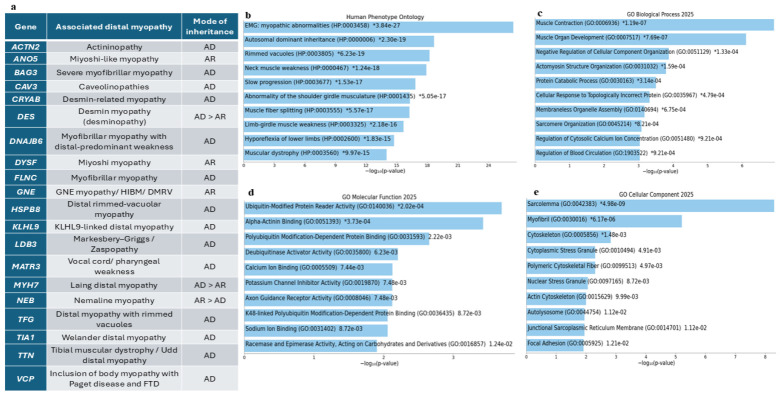
Genetic profile and ontology analysis of distal myopathies. (**a**) List of genes associated with various forms of distal myopathy, indicating their inheritance patterns. (**b**–**e**) Enrichment analysis of human ontology terms (**b**) and Gene Ontology (GO) categories: biological processes (**c**), molecular functions (**d**), and cellular components (**e**), and corresponding to the genes listed in (**a**). GO analyses were carried out with Enrichr tool: https://maayanlab.cloud/Enrichr/ (accessed on 16 July 2026).

To provide a comprehensive overview of the genetic landscape underlying distal myopathies, Figure 2 summarizes the 20 most common genes linked to distal myopathies, listing their modes of inheritance, and presenting an ontologicaly analysis based on these genes.

Ontology analysis revealed that some of the most common features associated with distal myopathies were abnormalities in electromyography (EMG) and the presence of rimmed vacuoles (Figure 2b). With respect to GO Biological Process (Figure 2c), the analyzed genes are predominantly involved in muscle contraction and muscle development. Consistently, GO Cellular Component analysis (Figure 2d) indicated that these genes are associated with key structural elements of muscle cells, including the sarcolemma, which constitutes the muscle cell membrane, and myofibrils, the contractile unit of muscle fibers. Notably, this category also highlights the formation of stress granules (SG). SG formation represents an adaptive cellular response to adverse conditions, as it entails the sequestration of specific messenger RNAs, non-coding RNAs (ncRNAs), and the resulting transient inhibition of translation, thereby reducing the protein synthesis burden during cellular stress. In agreement with these findings, GO Molecular Function analysis (Figure 2e) revealed that the analyzed genes are primarily associated with ubiquitination-related processes, a central regulatory system involved in the degradation of misfolded or damaged proteins. Taken together, these results underscore the importance of cellular stress response mechanisms in the pathophysiology of distal myopathies and highlight their relevance for future investigation.

### 1.1. Distal Myopathies Are a Heterogeneous Group

#### 1.1.1. Genetic Heterogeneity of Distal Myopathies

Distal myopathies are heterogeneous genetic disorders. Their prevalence indices vary owing to founder variants, which increase the prevalence of certain subtypes in specific communities [6,7,8]. A representative example is the higher incidence of WDM observed in the Baltic Sea region, particularly in Finland and Sweden. This disorder results from a pathogenic mutation in the TIA1 gene located on chromosome 2p13 [9,10]. Affected individuals from Sweden and Finland share the same pathogenic variant on a common ancestral haplotype, consistent with a founder effect estimated to have originated approximately 1050 years prior. Currently, no evidence supports recurrent de novo mutation as an underlying mechanism. Consequently, the prevalence of WDM in certain regions of Sweden is 1 in 10,000 [6,9,10], which is markedly higher than in other populations.

An increasing number of genes have been implicated in both autosomal-dominant and autosomal-recessive forms of distal myopathies [4]. Currently, approximately 25 genes are known to contribute to these disorders, and nearly 20 distinct forms have been described. These subtypes display marked heterogeneity in terms of clinical phenotype, distribution of muscle involvement, age at disease onset, and histopathological features [2].

A recent cohort study of 219 patients in Spain identified likely pathogenic variants in 68.7% of patients and showed that 85% of these variants were concentrated in eight genes: MYH7, anoctamin 5 (ANO5), dysferlin (DYSF), titin (TTN), myotilin (MYOT), heat shock protein family B (Small) member 1 (HSPB1), glucosamine (UDP-N-acetyl)-2-epimerase/N-acetylmannosamine kinase (GNE) and heterogeneous nuclear ribonucleoprotein D like (HNRNPDL) [11]. The first digenic form was recently described, involving the sequestosome 1 (SQSTM1) and TIA1 genes [12]. Moreover, certain genes (e.g., MYH7) have been shown to give rise to both autosomal dominant and autosomal recessive presentations, depending on the nature and location of the pathogenic variant [13].

#### 1.1.2. Clinical Heterogeneity and Phenotypic Presentation of Distal Myopathies

In general, all the diseases exhibit a slowly progressive course [2,14,15,16], although the rate of progression may vary depending on the variant’s localization within the gene [17]. Although the causative gene has been identified for each disease, the clinical phenotype may differ according to the specific mutation present in each patient [18]. Therefore, it is crucial to provide a detailed clinical and genetic description of individual cases to better understand genotype-phenotype correlations.

Among the assessments performed for diagnosis, measurement of creatine kinase (CK) levels is commonly included in the diagnostic process. In most cases, such as Udd distal myopathy (UDM), CK levels are within the normal range or mildly elevated [14]. However, certain distal myopathies, such as Nonaka myopathy (NDM), are associated with markedly elevated CK levels [15].

Distal myopathies can be distinguished based on the initial pattern of muscle involvement and the age onset. Dominant LIM domain binding 3 (LDB3)-related distal myopathy, also known as Markesbery-Griggs distal myopathy, usually begins with ankle weakness after 40 years of age [19]. Other distal myopathies with the same group of muscles involved are UDM and NDM.

UDM, initially reported in Finnish patients in 1993 [20], is characterized by selective weakness of the ankle dorsiflexor muscles, leading to foot drop and difficulty or inability to walk on heels. The onset of symptoms generally occurs after the age of 30 years [14].

NDM was originally described in Japan in the early 1980s [21]. Since then, it has been referred to by several names, including distal myopathy with rimmed vacuoles (DMRV) and hereditary inclusion body myopathy (hIBM), which were later unified as GNE myopathy [18]. The initial symptoms commonly include weakness affecting the ankle dorsiflexors [15].

In contrast, early onset distal myopathies include Miyoshi myopathy type 2 (MMD2) and Laing distal myopathy (MPD1). MMD2 typically begins during adolescence or early adulthood, leading to significant difficulties in standing on tiptoes or climbing stairs. The initial symptoms also include weakness of the calf and lower leg muscles [22]. MDP1 displays a variable onset and phenotypic severity [16]. It usually starts with weakness in the anterior compartment of the lower leg and progresses to other skeletal muscle groups, eventually involving the cardiac and respiratory muscles [16,17]. Cases have been reported in individuals ranging from 10 years of age to adulthood [17]. Table 1 provides a detailed comparison of these distal myopathy subtypes.

### 1.2. Clinical Tests for Myopathies in General

The measurement of serum CK is a simple, economical and common initial test for evaluating suspected myopathies [28]. It serves as a sensitive but nonspecific marker, where elevations usually indicate skeletal muscle damage, although they can also stem from cardiac injury or specific nerve disorders [29,30]. While marked hyperCKemia characterizes severe conditions such as Duchenne and Becker muscular dystrophies [31], other conditions such as WDM and milder limb-girdle muscular dystrophies may present with normal or mildly elevated CK levels [30]. This demonstrates that isolated hyperCKemia has limited predictive value on its own [32] and must be integrated into a multimodal diagnostic framework with clinical findings and electromyography (EMG) findings [33]. Within this framework, EMG plays a key role by providing invasive but valuable functional information about muscle fibers and motor units to confirm myopathic weakness and exclude neurogenic causes [34,35,36,37,38]. Complementing this, Magnetic Resonance Imaging (MRI) offers an essential, noninvasive, and comprehensive view of the entire musculoskeletal system, while neuromuscular ultrasound serves as a highly efficient, cost-effective, bedside alternative that readily detects dynamic changes, muscle atrophy, and increased echogenicity, such as the selective distal muscle involvement characteristic of WDM [33,34,35,36].

To secure a definitive diagnosis and evaluate disease progression or stage, a muscle biopsy remains crucial [39,40,41], although the sampled muscle must be clinically affected without reaching an end-stage or severely atrophied phase [39]. This tissue can be collected via an open muscle biopsy, which requires a 4–6 cm incision and risks complications such as hematoma, herniation, wound dehiscence, or infection [39], or through a less invasive needle muscle biopsy via a 4–5 mm incision [42], which is technically feasible for targeting the tibialis anterior muscle in distal myopathies, despite a smaller sample size [39]. Ultimately, despite clinical and genetic variations, muscular dystrophies evaluated using these methods share unifying histopathological findings, including fiber size variability, necrosis, macrophage infiltration, and progressive fibro-fatty replacement of the muscle tissue [43,44].

## 2. Overview of Welander Distal Myopathy

### 2.1. Epidemiology and Clinical Description

**Figure 3:**
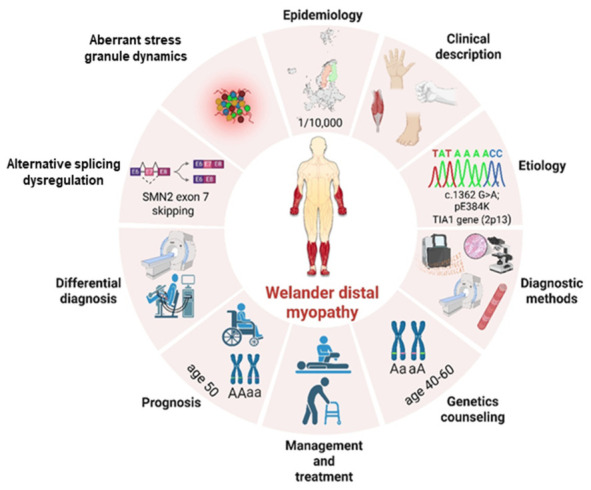
Comprehensive representation of Welander distal myopathy. The figure illustrates the estimated prevalence, characteristic clinical symptoms such as progressive distal muscle weakness, and the underlying genetic variant in the TIA1 gene (p.E384K). Diagnostic modalities, including genetic testing, imaging, and pathology, are presented alongside differential diagnoses. The prognosis, management options, and implications for genetic counselling are outlined. It also highlights the historical milestones related to the first descriptions of the genetic basis (i.e., etiology) and mechanistic aspects (i.e., molecular and cellular processes) associated with WDM [9,10]. This figure was created in BioRender. Author J.M.I. (4 June 2026).

Welander distal myopathy (WDM; OMIM: 604454) is an uncommon distal myopathy that predominantly affects the distal musculature of the upper limbs. It typically begins with weakness of the finger and wrist extensors and gradually progresses to involve all hand muscles (Figure 3). With disease progression, the distal muscles of the lower limbs may also become involved, particularly those responsible for toe and ankle extension [6,7,9,10].

Although the global prevalence remains unknown, WDM is mainly confined to regions surrounding the Baltic Sea, with a higher prevalence reported in Finland and central-eastern Sweden, where it affects approximately 1 in 10,000 individuals [9,10] (Figure 3). While most cases originate from this geographical area, additional patients have been identified in the United Kingdom and Germany. Moreover, a similar phenotype has been described in Spanish and French individuals with a digenic combination involving an SQSTM1/p62 mutation and TIA1-N357S variant. Familial segregation analysis confirmed this digenic model, as family members inheriting solely the TIA1 variant remained completely asymptomatic, proving that the myopathic phenotype strictly depends on the co-inheritance of both heterozygous mutations [45,46]. Based on these findings, some authors have proposed that although the p.E384K mutation in TIA1 is the most widely reported, TIA1 variants combined with other gene mutations may also contribute to WDM or related phenotypic presentations [19].

Clinically, WDM is considered a late-onset condition, with symptom onset typically occurring between 40 and 60 years of age. The disease initially presents with weakness in the index finger extensors, later progressing to involve other fingers and lower leg muscles (highlighted regions of the human body in Figure 3). The anterior tibial muscle and toe extensors are commonly affected, leading to walking difficulties and steppage gait. Proximal limb involvement is uncommon, and muscle stretch reflexes are usually preserved, except for the ankle reflex, which may diminish in later disease stages. No cardiac involvement was noted. Rare homozygous cases show an earlier onset, proximal muscle impairment, and rapid disease course [6,7,9,10].

### 2.2. Etiology

**Figure 4:**
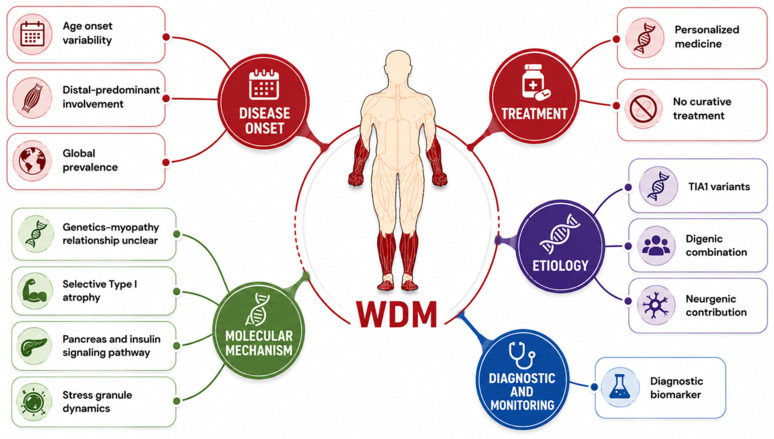
Conceptual overview of the aspects under investigation of WDM. Key domains include disease onset, treatment, etiology, molecular mechanisms, and diagnostic and monitoring approaches, emphasizing current knowledge gaps and ongoing research directions in the field. This figure was modified using ChatGPT Free (https://chatgpt.com/de-DE/plans/free/, accessed on 16 July 2026).

The molecular basis of WDM is highly uniform and traced to a specific heterozygous missense founder mutation in the TIA1 gene (c.1362G > A; p.E384K), located on chromosome 2p13 [9,10]. This specific mutation is highly conserved across Nordic populations, particularly within Swedish and Finnish cohorts, indicating a distinct dominant-negative or toxic gain-of-function mechanism unique to this single amino acid substitution. WDM follows an autosomal dominant inheritance pattern with complete penetrance by age 75, making genetic counselling an important component of care [7,9,10] (Figure 3). In addition, the WDM phenotype has been reported in Spanish and French patients with a digenic combination of an SQSTM1/p62 mutation and TIA1-N357S variant [45,46] (Figure 4).

### 2.3. Diagnostic Methods

In modern clinical neuro-muscular-genetics, the diagnostic landscape for WDM has undergone a significant paradigm shift. Historically, the diagnostic pathway relied heavily on a combination of clinical phenotyping, electrophysiological studies, and invasive muscle biopsies. Today, however, diagnosis primarily relies on molecular genetic testing as the definitive, first-line diagnostic standard.

Given that WDM is a highly penetrant, autosomal dominant disorder caused by a specific heterozygous missense mutation in the TIA1 gene (specifically the founder mutation c.1362G > A; p.E384K), targeted genetic sequencing or multi-gene panels offer near-perfect diagnostic reliability. Identifying this mutation provides an unequivocal diagnosis, bypassing the need for more invasive or less specific diagnostic procedures. Indeed, in current clinical practice, many patients are diagnosed directly via molecular genetics, effectively bypassing electrodiagnostic testing or muscle biopsies altogether.

Despite the paramount role of genetic testing, traditional evaluative methods retain important supportive and historical value, particularly in characterizing disease progression or when genetic access is limited. For instances, serum CK levels typically remain normal or only slightly elevated, helping to distinguish WDM from more aggressive, highly dystrophic myopathies. EMG characteristically reveals a classic myopathic pattern in affected muscles, typically manifested as short-duration, small-amplitude, and polyphasic motor unit action potentials, often accompanied by spontaneous activity such as fibrillation potentials and positive sharp waves in the more rapidly progressing phases. Electromyographic studies usually reveal myopathic motor units, sometimes mixed with neuropathic features, and may include fibrillations or complex repetitive discharges. Muscle imaging often demonstrates fatty degeneration in posterior calf muscles and involvement of the anterior muscle compartment [6,9,10] (Figure 3). MRI analysis in WDM can illustrate early fatty degeneration of hand extensors, progressing to the anterior compartment of the lower leg (tibialis anterior) [15,19].

Neuromuscular ultrasound is a powerful, noninvasive diagnostic tool. Ultrasound readily detects increased muscle echogenicity (due to fibro-fatty replacement) and muscle atrophy, showing in particular a distinctive pattern of selective muscle involvement in the distal extremities (extensors of the hands and feet), consistent with the clinical presentation of WDM. Compared to MRI, this diagnostic tool is a cost-effective, efficient, noninvasive, and dynamic option that can be used at the bedside (Figure 3) [15,19].

Histopathological analysis of WDM reveals selective atrophy of Type I muscle fibers and myopathic changes with rimmed vacuoles in distal muscles [47]. Biopsy studies demonstrated an irregular distribution of intramuscular fat and abnormal formazan staining, indicative of altered oxidative enzyme activity, which are early hallmarks of atrophic Type I fibers. In contrast, fibers exhibiting normal size and enzyme distribution were predominantly lightly stained, consistent with Type II fibers [47]. Furthermore, examinations of affected muscles indicated a lack of low-frequency motor units, corresponding to the typical organization of Type I fibers into motor units with low discharge frequencies [47].

These observations underscore the presence of selective Type I fiber atrophy (Figure 3), accompanied by disrupted oxidative enzyme patterns and abnormal lipid deposition. In addition, the altered firing patterns of innervating motoneurons in distal myopathy [48] suggest a neurogenic contribution to the muscle fiber pathology in WDM [47,49] (Figure 4). Therefore, while these traditional phenotypic markers remain essential for understanding the underlying muscle pathophysiology and assessing functional decline, they also serve primarily to support the definitive molecular diagnosis.

### 2.4. Genetic Counseling

For families carrying the TIA1 gene mutation, genetic counseling serves as an essential compass for navigating the autosomal dominant nature of WDM, which carries a 50% chance of transmission to offspring (Figure 3). Because the condition typically remains silent until after age 40, gradually impairing fine motor control in the hands and feet, counselors provide vital support for asymptomatic relatives weighing the psychological and practical decision to undergo predictive testing [50]. Crucially, counselors clarify how the genetic “dose” dictates disease severity: a single mutated copy (i.e., heterozygosity) generally leads to a slow-progressing, manageable course, whereas the rare inheritance of two mutated copies (i.e., homozygosity) results in a highly aggressive, early-onset phenotype. By exploring family planning options like pre-implantation genetic testing and coordinating early, proactive physical therapy, genetic counseling transforms a daunting genetic legacy into an empowered, manageable path forward [19,50] (Figure 3 and Figure 4).

### 2.5. Managements and Treatment

No curative treatment is currently available for WDM (Figure 3), and management is focused on symptomatic support aimed to preserve function and quality of life [9,10]. Physical and occupational therapy, adaptive tools for hand function, and orthoses for wrist weakness or foot drop may help maintain autonomy and mobility [9,10,51]. For example, ankle-foot orthoses are used to manage foot drop and improve balance [52] (Figure 3 and Figure 4).

### 2.6. Prognosis and Differential Diagnosis

The disease generally progresses slowly, and although life expectancy remains normal, patients often lose fine hand motor skills due to the disease. Accordingly, it should be noted that a homozygous genotype exhibits a more severe clinical course and any person with this may require wheelchair assistance by around 50 years of age [8,9] (Figure 3). Muscle biopsies from patients with WDM reveal classic myopathic features, including marked fiber size variability, split fibers, and internally placed nuclei. The diagnostic hallmark is the presence of rimmed vacuoles and invaginations of the sarcolemma, lined with autophagic debris. Additionally, affected myofibers demonstrate abnormal cytoplasmic protein aggregations containing TIA1, TIA1-related/like protein (TIAR/TIAL1), TARBP/TDP-43, SQSTM1/p62, ubiquitin, etc., linking the condition directly to systemic proteinopathies (Figure 4) [9,10].

### 2.7. Pathophysiological Cellular and Molecular Basis

The first pathophysiological evidence involving cellular and molecular investigations [9,10] indicated that the E384K TIA1 mutation could disrupt SG dynamics under stress conditions [9] and alternative splicing events involving exon 7 skipping of the survival motor neuron 2 (SMN2) gene [10] (Figure 3 and Figure 4). Emerging cellular models with this mutation have elucidated how oxidative and other types of stress contribute to disease progression [9,10,53,54,55,56,57,58]. Although recent research in cellular and molecular biology has explored the broader systemic implications of the TIA1 gene mutation that causes WDM, a clear discrepancy remains between in vitro models and the historical clinical phenotypes. In this regards, the missense E384K TIA1 autosomal dominant mutation has been reported to affect β-cell insulin production and viability. The E384K TIA1 variant caused major dysfunction when introduced into human insulin-producing EndoC-βH1 cells by CRISPR-Cas9 gene editing. The mutated cells showed higher MYC mRNA and protein levels, profoundly reduced GLP-1 receptor mRNA expression, increased expression of “disallowed” β-cell genes, proinsulin-to-insulin processing defects, decreased insulin content and release, decreased PAX4/ARX mRNA ratio, and increased glucagon production. In other words, the E384K TIA1 mutation that destroys distal muscle function simultaneously causes β-cell dedifferentiation, pushing β-cells away from their insulin-producing identity and toward a glucagon-producing, less mature state [59]. This recent study suggested that the TIA1 gene mutation associated with WDM can induce pancreatic beta-cell dysfunction through cellular dedifferentiation, in which the cells lose their mature identity and cease to produce insulin without undergoing apoptosis [59]. Notably, this experimental condition proved to be reversible, and treatment with a GLP-1 receptor agonist successfully restored both the identity of mature beta cells and the proper mechanics of insulin secretion in vitro [59]. This is clinically important since it raises the possibility that WDM patients with the E384K mutation may have subclinical or overt pancreatic endocrine dysfunction, potentially contributing to diabetes, and that GLP-1 receptor agonists (already widely used for diabetes) could partially rescue this β-cell defect (Figure 4).

## 3. T-Cell Intracellular Antigen 1 (TIA1)

**Figure 5:**
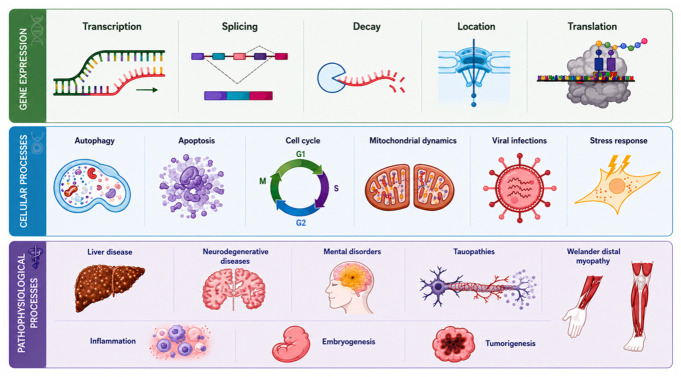
Summary of the biological processes associated with TIA1. The figure is divided into three panels detailing the mechanisms of gene expression (**top**), cellular processes (**middle**) and physiological and pathological conditions (**bottom**) in which TIA1 is involved. This figure was modified using ChatGPT Free (https://chatgpt.com/de-DE/plans/free/, accessed on 16 July 2026).

TIA1 is an RBP that plays a central role in multiple processes related to RNA metabolism and the regulation of gene expression [60,61,62,63,64,65,66,67,68,69,70,71,72,73,74,75,76,77,78,79,80]. Although initially identified in the immune system, it is also expressed in multiple cell types and tissues [62,73,80] (Figure 5).

### 3.1. TIA1 Gene and Protein Structure

**Figure 6:**
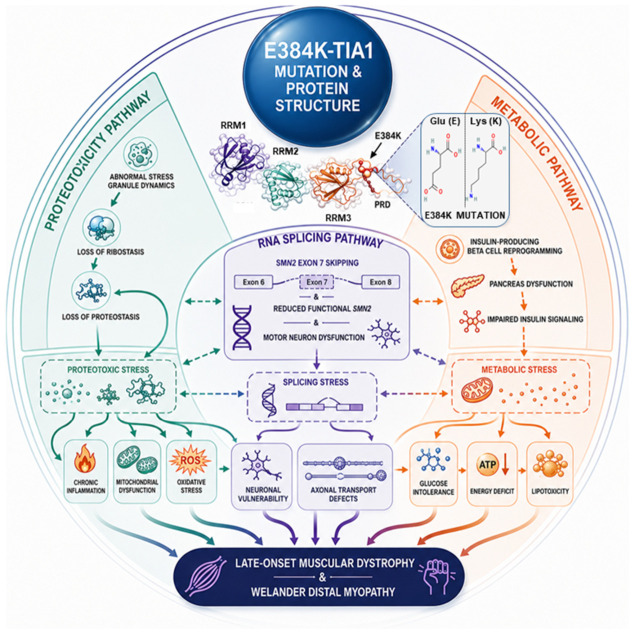
Potential pathophysiological overview of the consequences of the E384K mutation in the TIA1 gene: molecular cascades that cause proteotoxic, ribotoxic/splicing, and metabolic stress, leading to the development of Welander distal myopathy (WDM). This figure outlines the multilayered pathogenic cascades induced by the E384K-TIA1 mutation, detailing how a single genetic alteration propagates into systemic myodegeneration. At the structural level, the TIA1 protein is composed of three RNA Recognition Motifs (RRM1-RRM3) and a C-terminal prion-related domain (PRD). The E384K missense mutation, situated within the PRD, replaces a negatively charged glutamate (E) with positively charged lysine (K), disrupting the conformation and molecular dynamics. This structural alteration triggers three distinct primary pathological axes: abnormal SG dynamics coupled with a loss of ribostasis and proteostasis; RNA splicing defects characterized by survival motor neuron 2 (SMN2) exon 7 skipping, leading to reduced functional SMN2 and subsequent potential motor neuron dysfunction; and pancreatic beta-cell reprogramming, which results in pancreatic dysfunction and impaired insulin signaling. As these primary defects progress, they converge into three highly interconnected cellular stress phenotypes: proteotoxic, ribotoxic, and metabolic stress. Extensive molecular crosstalk between these stress modules further exacerbates cellular injury. Downstream, proteotoxic and splicing/ribotoxic stresses may promote chronic inflammation, mitochondrial dysfunction, oxidative stress, neuronal vulnerability, and axonal transport defects, whereas metabolic stress concurrently drives glucose intolerance, energy deficit through decreased ATP production, and lipotoxicity. Ultimately, these overlapping degenerative mechanisms directly affect and converge to contribute to the clinical presentation of WDM. (Note: Solid arrows indicate direct effects; dashed arrows indicate molecular crosstalk; color-gradient arrows represent converging contributions). This figure was modified using ChatGPT Free (https://chatgpt.com/de-DE/plans/free/, accessed on 16 July 2026).

The TIA1 gene produces two main mRNA and protein isoforms, TIA1a and TIA1b, through alternative splicing of exon 5, which may be either included or excluded. Exon 5 generates an additional peptide segment of 11 amino acids, giving rise to the TIA1a variant, thereby differentiating it from the TIA1b isoform [61,62]. This exon is highly conserved between humans and mice [61,62], and the resulting isoforms are differentially expressed depending on the cellular and tissue context [62,73,74,75,76,77]. Structurally, TIA1 conforms to the canonical architecture of RBPs, containing three RNA-recognition motifs (RRM1–3) and a C-terminal region enriched in glutamine and asparagine residues (Q/N-rich domain) [62,76]. This region is also known as prion-related/like domain (PRD) [76,79]. Regarding its RRM domains, RRM2 and RRM3 exhibit the highest degree of evolutionary conservation in vertebrates and play a predominant role in RNA interaction and sequence recognition [62,76]. The Q/N domain is characterized by intrinsically disordered low-complexity sequences that mediate protein–protein interactions together with atypical RRM1 [64,65,66,67,70] (Figure 5 and Figure 6).

### 3.2. The Function of TIA1 and Associated Pathological Events

TIA1 functions as a central coordinator of communication between the nuclear and cytoplasmic compartments in eukaryotic cells, exerting broad regulatory effects on RNA metabolism [62,63,64,65,66,67,68,69,70,71,72,73,74,75,76,77,78]. It participates in multiple stages of post-transcriptional gene regulation, including both constitutive and alternative splicing [68,69,70,71,75,76,77,78], as well as in the intracellular trafficking and localization of mRNAs [65,66,67]. In addition, TIA1 contributes to the control of (m)RNA stability and translation [63,64,65,66,67,72,73] by directly binding to RNA molecules or competing with other RBPs and regulatory factors [71,72,73,75,76,77,78] (Figure 5).

At the molecular level, TIA1 preferentially binds U-rich, UC-rich, and AU-rich sequence elements present in pre-(m)RNAs and mature transcripts. These motifs are frequently located near suboptimal splice sites within introns, as well as within the 5′ and 3′ untranslated regions of mRNAs, and can also be found in non-coding RNAs [63,75,76,77,78]. Genome-wide analyses suggest that a substantial proportion of human transcripts, potentially ranging from 5% to 10%, are targets of TIA1-mediated regulation [75,76,77,78].

By selectively recognizing these sequence elements, TIA1 influences splice site selection, thereby modulating exon inclusion or exclusion. This regulatory capacity has significant consequences for alternative splicing patterns and ultimately contributes to the diversification of transcriptomes and proteomes within the cell [75,77,78].

Alterations in TIA1 expression levels and/or its intracellular distribution are associated with a wide spectrum of biological processes and pathological conditions. These include roles in early developmental events, such as embryogenesis, as well as inflammation [64,71,81,82,83,84], tumorigenesis [85,86,87,88,89,90], neuronal homeostasis [91,92,93], and various neuromuscular and neurodegenerative disorders, including tau-related pathologies [93] and myopathies [9,10,12,53,54,55,56,57,58,59]. In addition, TIA1 is implicated in cellular stress responses [65,66,67] and host–virus interactions [80,94] (Figure 5).

The diversity of these associations reflects the extensive involvement of TIA1 in regulating various cellular pathways. These include programmed cell death [60,72,87,88,89], autophagic and mitophagic processes [81,95,96,97], cellular senescence, and immune system modulation [64,73,82,83,84]. TIA1 also contributes to membrane organization [98], axonal repair mechanisms [99], and the control of protein synthesis through its effects on translational machinery activity and localization [100,101]. Further functions include the modulation of the cell cycle [85,86,87,88,89,90], mitochondrial dynamics [95,96,97], and maintenance of proteostasis [102,103] (Figure 4).

Consistent with this broad functional involvement, evidence from animal models underscores the biological relevance of TIA1 in various diseases. Genetic ablation in mice results in substantial embryonic lethality [64], although the degree of penetrance is influenced by the genetic background of mice [64,83,84]. Surviving adult knockout animals show a mild-arthritis phenotype [64] and exhibit behavioral and molecular features reminiscent of chronic stress-related disorders in humans, with a more pronounced effect in females [91]. Moreover, TIA1 haploinsufficiency aggravates neuroinflammatory responses in models of tau-associated neurodegeneration [93,104], further highlighting its relevance in the contexts of disease.

At the molecular level, TIA1 is widely recognized for its role in translational control and formation of SG. Under conditions that inhibit translation, mRNAs released from polysomes assemble into two principal types of cytoplasmic RNA granules in mammalian cells: processing bodies (P-bodies) and SG [57,65,66,67,105,106,107], which function as key post-transcriptional regulators of gene expression (i.e., cellular ribostasis and proteostasis).

### 3.3. Importance of TIA1 in SG Dynamics

SGs are riboprotein complexes composed of more than 450 proteins and 11,000 transcripts (https://rnagranuledb.lunenfeld.ca) (accessed on 4 June 2026). As an adaptive survival response to acute stress, they prevent cell death by temporarily pausing protein synthesis, shielding mRNAs from degradation, and prioritizing the translation of critical stress-response and housekeeping genes. This mechanism ultimately protects cellular health by reducing the load on protein folding and synthesis machinery to maintain ribostasis and proteostasis [65,66,67,106,107,108,109,110].

The assembly of SG is commonly initiated by the activation of the integrated stress response (ISR) [110,111], which converges on phosphorylation of the α subunit of eukaryotic initiation factor 2 (eIF2α) by one of four stress-sensing kinases (i.e., heme-regulated inhibitor (HRI) kinase, protein kinase R (PKR), general control nonderepressible 2 (GCN2) kinase, and endoplasmic reticulum (ER)-resident kinase (PERK)). Phosphorylated eIF2α inhibits the formation of the eIF2–GTP–Met-tRNAi ternary complex, thereby blocking translation initiation and promoting global polysome run-off with the release of large amounts of non-translating mRNAs into the cytosol [110,111]. These untranslated mRNAs, along with translation initiation factors, serve as seeds to trigger the assembly of SGs [110,111]. This allows for rapid translational reprogramming and ensures an efficient recovery once the cellular stress subsides.

The TIA1 protein, alongside Ras-GTPase-activating protein-binding protein 1/2 (G3BP1/2), is essential for seeding the assembly and regulating the behavior of cytoplasmic SGs [65,66,67,106,107,108,109,110]. While TIA1 resides mostly in the nucleus under normal conditions, it continuously shuttles between the nucleus and the cytoplasm [65]. In response to stress, it accumulates in the cytoplasm and promotes the recruitment of untranslated (m)RNAs and proteins to SGs [66,67]. These compartments function as interactive platforms where coding and non-coding RNAs aggregate and organize specific RBPs and other non-RBPs. By clustering these molecules closer together, they facilitate phase separation from an energetic standpoint. This process ultimately determines how these stress-responsive RNA-protein granules form, how they remain stable, and how they eventually disintegrate [65,66,67,106,107,108,109,110].

SG disassembly is generally slower and mechanistically more heterogeneous than assembly [112]. Upon stress removal, SGs fragment into smaller foci that dissolve individually, consistent with the stepwise reversal of the multivalent protein–RNA interaction network [113]. The gradual restoration of SG dissolution is a primary driver of translation. As eIF2α is dephosphorylated through the activating transcription factor 4 (ATF4)–protein phosphatase 1 regulatory subunit 15A (PPP1R15A/GADD34) feedback axis [114], ribosomes reload onto mRNAs, reducing the pool of ribosome-free transcripts that sustain SG integrity [65]. However, polysome recovery may lag behind the disappearance of microscopically visible SGs, indicating that additional active clearance pathways operate in parallel [115]. Several ATP-dependent machineries contribute to SG clearance, including the AAA+ ATPase valosin containing protein (VCP/p97) and its unc-51 like autophagy activating kinase ½ (ULK1/2)-dependent phosphorylation [116], as well as autophagy-related proteins such as C9orf72-SMCR8 complex subunit (C9orf72) and SQSTM1 [117]. Molecular chaperone systems, including the heat shock protein family B (small) member 8 (HSPB8)–BAG cochaperone 3 (BAG3)–heat shock protein family A (Hsp70) member 1A (HSP70) complex, function as surveillance mechanisms to prevent aberrant liquid-to-solid transitions driven by misfolded proteins within SGs [118].

Alterations in SG dynamics can have pathological consequences. The PRD of TIA1 mediates its aggregation capacity, and the WDM-associated mutation p.E384K located within this domain replaces a conserved glutamate with lysine, reversing local charge and likely altering domain functionality [9,53,54,55,56,57,58]. Cells expressing mutant TIA1 displayed a mild but reproducible increase in SG number and size under stress compared to wild-type cells. Furthermore, the mutation slows TIA1 exchange between SGs and the cytosol, indicating defective granule remodeling and turnover [9,53,54,55,56,57,58] (Figure 4). Over time, this increased aggregation propensity may favor the persistence of aberrant SG remnants in skeletal muscle and promote secondary protein aggregation, ultimately overwhelming the cellular protein-quality control systems [9].

TIA1 function is not only perturbed by the E384K substitution; additional mutations within PRD have been identified that similarly promote SG assembly and are linked to neurodegenerative disorders, such as amyotrophic lateral sclerosis (ALS). Notably, the A381T variant has been shown to enhance TIA1 self-assembly by stabilizing β-sheet interactions, thereby favoring the formation of irreversible amyloid fibrils and aberrant SGs [53,119,120]. Likewise, the P362L substitution within the PRD increases the propensity of TIA1 to undergo phase separation, resulting in the persistence of SGs and defective granule disassembly dynamics [53].

SGs not only function as repositories for translationally stalled mRNAs but also actively modulate cellular responses by selectively sequestering critical transcripts, thereby promoting downstream processes, such as inflammation. For example, in cerebral ischemia models, TIA1 drives pro-inflammatory microglial activation by sequestering insuli-like growth factor 2 (IGF2) mRNA into SG, resulting in attenuation of IGF2-mediated anti-inflammatory signaling [121,122]. This mechanism, characterized in microglial cells, may similarly contribute to muscle degeneration in patients with Welander myopathy [112].

WDM represents a localized failure of protein homeostasis and RNA metabolism driven by the structural disruption in TIA1, a ubiquitously expressed master regulator of post-transcriptional gene expression. Structurally, TIA1 features three RRMs and a C-terminal domain that resembles a PRD. Functionally, it splits its duties between the nucleus, where it regulates alternative splicing, and the cytoplasm, where it acts as a primary scaffold for SGs to halt non-essential translation during periods of cellular stress. The defining paradox of WDM pathogenesis is why the ubiquitous TIA1 E384K mutation triggers an isolated, late-onset myopathy rather than a systemic disorder, a tissue specificity governed by a cascade where the mutated WDM-TIA1 within the skeletal muscle environment leads to impaired SG clearance, formation of rimmed vacuoles, and ultimate myopathy. This muscle-specific degeneration may occur because permanent post-mitotic skeletal muscle fibers are incapable of diluting toxic protein states through cell division. The WDM-TIA1 mutation alters the biophysical properties of the TIA1 PRD, delaying SG dissolution and driving abnormal liquid-to-solid phase transitions that accumulate over decades into semi-irreversible and toxic aggregates. This proteotoxic kinetic vulnerability is compounded by the continuous mechanical strain and high metabolic turnover inherent to skeletal muscle, which chronically provokes physiological stress and recurring SG assembly, ultimately overwhelming local autophagic clearance mechanisms. Finally, the pathophysiology may be restricted to muscle tissue due to a unique local RNA-RNA, RNA-protein and/or protein-protein interactome. Unlike non-muscle tissues, which deploy robust heat shock proteins and clearers, such as valosin-containing protein and SQSTM1/p62, to degrade delayed SGs, the skeletal muscle environment fails to clear the mutated TIA1 complex. Consequently, essential muscle-specific (m)RNA transcripts encoding vital sarcomeric or sarcoplasmic reticulum proteins become chronically sequestered within these non-functional WDM SG, causing a localized loss-of-function phenotype that culminates in myofiber degeneration and rimmed vacuole formation (Figure 6).

### 3.4. New Insights About TIA1 Function

In addition to its well-established cellular functions, TIA1 has recently been implicated in several liver pathologies via novel molecular interactions. One notable study investigated the relationship between TIA1 and the pre-mRNA processing factor kinase PRP4K (PRPF4B) in hepatocellular carcinoma (HCC) [123]. PRPF4B, a kinase involved in the regulation of pre-mRNA splicing, modulates alternative splicing of TIA1 by promoting exclusion of exon 5. This splicing event suppresses the activation of the NF-κB inflammatory signaling pathway, ultimately contributing to an antitumoral effect [123].

These findings extend beyond liver cancer and may also be relevant to neurodegenerative and muscular disorders associated with protein aggregation. In this context, PRPF4B could represent a potential pharmacological regulator capable of modulating TIA1 behavior and its pathogenic aggregation properties.

Additional evidence supporting the relevance of TIA1 in liver disease has emerged from studies on metabolic dysfunction-associated fatty liver disease. In this setting, TIA1 appears to exert a protective role against hepatic metabolic stress through SGs formation [124]. Mechanistically, TIA1 directly binds to the mRNA of sterol regulatory element binding transcription factor 1 (SREBF1) and sequesters it within SGs. This process reduces translation and facilitates mRNA degradation, thereby limiting lipid accumulation and attenuating hepatic inflammation [124].

Similarly, a recent study addressed how a specific genetic mutation in WDM-TIA1 causes diabetes by making pancreatic beta cells (EndoC-βH1) lose their identity and stop producing insulin (dedifferentiation), rather than dying. This study demonstrated that cellular dysfunction is not permanent. By treating these cells with a GLP-1 receptor agonist, researchers successfully reversed the process, restoring the mature identity of the cells and their ability to secrete insulin [59]. However, to date, WDM remains poorly investigated and underdocumented from clinical and biomedical perspectives, and no study to date has examined WDM patients for fasting glucose/HbA1c profiles, glucose tolerance tests, fasting insulin and C-peptide levels, glucagon-to-insulin ratios, β-cell imaging or function testing, insulin signaling pathway biomarkers (IRS-1, Akt phosphorylation, etc.) in muscle biopsies. In 2026, this represents an important and open research question that further investigations urgently need to address. The WDM patient cohorts in Sweden and Finland, where the TIA1 E384K founder mutation has been known for decades, provide an ideal population in which to conduct the first systematic endocrine and metabolic assessments. In summary, the bibliographic and medical literature does not contain any data on glucose metabolism or insulin signaling in patients with WDM. However, the molecular and cellular evidence potentially predicts that such abnormalities exist, but their clinical validation remains to be performed (Figure 6).

## 4. Future Perspectives

### 4.1. In Vitro Cellular Models

The current understanding of WDM derives from a limited number of patient muscle biopsies and, given the difficulty in accessing such samples, also rests largely on knowledge gleaned from heterologous cellular systems [9,10,12,53,54,55,56,57,58,59]. These experimental approaches have been instrumental in establishing a mechanistic link between disease-causing mutation and altered cellular dynamics. However, heterologous cell models have limitations, as the cell type itself could play a relevant role in sensitivity to the TIA1 E384K mutation [9,10,12,53,54,55,56,57,58,59]. Therefore, their reliance on cellular context limits their physiological relevance. Given that WDM is a skeletal muscle–restricted disorder, models lacking the transcriptional, metabolic, and structural landscapes of differentiated myofibers are unlikely to fully recapitulate the disease-specific mechanisms. More broadly, concerns regarding the reproducibility and translational validity of preclinical models have been widely discussed in biomedical research, underscoring the importance of standardized and biologically appropriate experimental systems [125,126]. In the context of WDM, the absence of a unified, genetically defined myogenic cellular platform represents a critical gap that may hinder cross-study comparability and the mechanistic clarity of the results. Although TIA1 is ubiquitously expressed, WDM is clinically restricted mainly to skeletal muscles. Therefore, the muscle-specific context is likely to be critical for understanding the mechanisms of the disease. Skeletal muscle cells exhibit unique metabolic demands, mechanical stress exposure, and differentiation-dependent RNA regulation [127]. Thus, studying mutant TIA1 in non-muscle cells may obscure muscle-specific pathogenic mechanisms (Figure 5 and Figure 6).

A standardized human myoblast model harboring a knock-in TIA1 mutation would significantly enhance reproducibility and cross-laboratory comparability of these results. Moreover, there are reports of WDM patients with additional mutations involving MYH7 [128] or SQSTM1 [14,31], which accentuate the phenotypes and complex pathophysiological contexts of the disease. Human induced pluripotent stem cell (hiPSC)-based models offers a promising avenue for overcoming these limitations. Reprogramming technologies allow the generation of patient-specific pluripotent lines that retain these pathogenic mutations [129]. Directed differentiation protocols now enable the efficient generation of skeletal myogenic progenitors and contractile myotubes from hiPSC through the staged activation of developmental signaling pathways, including Wnt family member 3 (WNT) activation and BMP-binding endothelial regulator (BMP) inhibition [130]. Alternative strategies employing inducible MyoD family inhibitor (MYOD) overexpression have also demonstrated robust myogenic conversion from pluripotent cells [131,132]. These approaches provide a muscle-relevant system in which TIA1 function can be interrogated at the endogenous expression levels. These properties could help advance the development of models of human muscle diseases, which, in the long term, would lead to a better understanding of how this disease progresses in people.

Nevertheless, the ultra-rare nature of WDM presents practical challenges for patient-derived modeling. The disease has been primarily described in Swedish families carrying a founder mutation [6,9,10], limiting access to biological material. In this context, CRISPR/Cas9 genome editing offers a complementary strategy to introduce the TIA1 E384K mutation into control of pluripotent stem cells derived from healthy donors, generating isogenic mutant–control pairs [133].

Beyond genetically defined 2D myogenic systems, three-dimensional (3D) engineered skeletal muscle constructs provide a more physiologically relevant microenvironment, allowing myofibers to align, remodel their extracellular matrix, and develop more mature contractile and metabolic properties than in 2D monolayers (e.g., enhanced mitochondrial maturation, ECM organization, and fast-twitch signatures) [134,135]. More broadly, transitioning from planar cultures to 3D tissues has been advocated as a critical step to better recapitulate in vivo–like signaling gradients, mechanical constraints, and multicellular architecture, thereby improving the predictive value of in vitro models [136].

In addition to structural maturation, the integration of multiple cell types is essential to address the questions regarding the pathophysiology of WDM. Although WDM is classified as a primary distal myopathy, early clinical and morphological studies suggested a potential neurogenic component. Histochemical and neurophysiological observations in Swedish cohorts identified signs of terminal motor neuron involvement, including fiber-type grouping and reduced nerve conduction amplitudes [137,138]. These findings raise the possibility that TIA1 mutations impair the maintenance of the neuromuscular junction (NMJ) or affect bidirectional signaling between motor neurons and muscle fibers.

In WDM, the founder E384K mutation in TIA1 leads to distal muscle weakness with accumulation of P-bodies and SG-associated proteins into rimmed vacuoles in muscle [9,10]. TIA1 mutations have also been firmly linked to other neuromuscular diseases, including their association with motor neuron degeneration in amyotrophic lateral sclerosis (ALS) [53,119,120]. This dual distribution of pathology suggests the possible involvement of shared processes between both compartments [139,140].

Recent studies have identified a role for TIA1 in the axonal transport of ribonucleoprotein complexes, highlighting the function of annexin A7 (ANXA7) in retrograde transport toward the neuronal soma [140]. Disruption of this process leads to pathological accumulation of TIA1 in axons, which could compromise functions dependent on axonal transport, including maintenance of the neuromuscular junction (NMJ). However, the mechanisms linking TIA1 dysfunction to impaired communication between motor neuron and muscle fiber remain unclear. The available evidence is largely limited to intracellular mechanisms, such as altered SG dynamics, aberrant phase separation, and mRNA sequestration [140]. Currently, there are no direct data demonstrating that TIA1 mutations affect bidirectional signaling at the NMJ.

As a future direction in this field, neuromuscular organoids (NMOs) would represent an interesting approach to study the molecular basis of this disease [141], as the motor unit can be established. Recent innovations in skeletal muscle organoids have highlighted their potential for mimicking complex neuromuscular circuits and multi-tissue interactions. In fact, established protocols have demonstrated the ability to co-differentiate spinal cord neurons and skeletal muscle cells within a single, self-organizing organoid from iPSC, creating a holistic model of motor units [142]. These NMOs are complemented by refined bioengineering approaches that employ microstructured devices or 3D hydrogels to facilitate the interaction between independently derived cell populations. In these systems, iPSC-derived motor neurons extend axons to innervate human myofibers, resulting in contractile tissues in which neuromuscular transmission, synaptic morphology, and axonal outgrowth can be quantitatively assessed [143]. Refined rapid assembly models have further shown that functional human NMJ-like tissues can be established within days, displaying robust acetylcholine receptor (AChR) clustering and neuron-dependent muscle contractions [144]. It should be noted that the development of organoids still has key challenges limiting their broader biomedical applications, including difficulties in recapitulating dynamic cell–cell and cell-extracellular matrix interactions [141].

Access to cellular samples from biobanks would be advantageous for developing a cellular model. Disease-specific biobanks play a critical role in advancing research on rare diseases, where limited patient numbers and fragmented datasets often hinder mechanistic and translational studies. By collecting and distributing well-characterized biological samples linked to clinical and genetic information, these infrastructures facilitate collaboration and enable access to patient-derived material that are otherwise difficult to obtain [145]. In the neuromuscular field, the MRC Centre for Neuromuscular Diseases Biobank represents a key initiative, providing a centralized repository of DNA, muscle biopsies, fibroblasts, and other patient samples for research [146]. Such coordinated biobanking efforts are particularly valuable for ultra-rare conditions, such as WDM, where access to patient material may significantly accelerate disease modelling and therapeutic research [147].

### 4.2. In Vivo Animal Models

Progress in WDM research is limited by the absence of an in vivo model that faithfully reproduces the TIA1 E384K mutation and late-onset distal muscle phenotype. While cellular systems have provided valuable mechanistic insight, they cannot fully capture organism-level processes, such as systemic metabolism, mechanical load, tissue interactions, and aging, all of which may influence disease onset and progression.

The importance of suitable animal models is illustrated by studies on other myopathies in humans. In GNE myopathy, genetically engineered mouse models carrying disease-associated mutations have been instrumental in clarifying the role of impaired sialic acid biosynthesis and have enabled preclinical evaluation of therapeutic strategies aimed at restoring sialylation, including supplementation approaches and other disease-modifying interventions [148]. Similarly, genome-engineered models of MYH7-related distal myopathies have provided important insight into in vivo sarcomeric dysfunction. For example, a CRISPR-engineered *Drosophila* model carrying the recurrent MYH7 K1729del mutation reproduces structural muscle defects and impaired motor function, demonstrating how genetically faithful models can recapitulate key pathological features and facilitate identification of potential therapeutic targets [149]. Together, these examples highlight the central role of disease-specific animal models in bridging molecular discoveries and translational research.

In this context, to elucidate the pathophysiological aspects associated with this mutation, and given the phylogenetic impossibility of generating models in invertebrates (i.e., flies and worms), the development of a humanized mouse with the Tia1 E384K knock-in mutation may represent a crucial step forward in WDM research. Because WDM is inherited in an autosomal dominant manner, a heterozygous knock-in model closely mimics the genetic condition observed in patients. Establishing a Tia1 E384K mouse model using CRISPR-based genome editing would provide a physiologically relevant platform for investigating the in vivo consequences of the mutation, including its effects on muscle physiology, SG dynamics, pancreatic dysfunction, and age-dependent disease progression. In addition to standard CRISPR/Cas9 approaches, emerging technologies, such as adenine base editing (ABE), may further improve the precision of model generation. ABEs enable direct A·T–G·C base conversion without introducing double-strand breaks, making them particularly suitable for introducing single-nucleotide variants, such as the c.1362G > A substitution underlying the Tia1 E384K mutation [150].

However, because WDM is a late-onset disorder, a simple knock-in model may not develop a detectable phenotype within the ~1-year lifespan of a mouse. In several neuromuscular disease models, physiological stress paradigms, such as exercise or oxidative stress, have been used to unmask or accelerate muscle pathology. For instance, exercise exacerbates muscle damage and oxidative stress in the mdx mouse model of Duchenne muscular dystrophy, facilitating the detection of disease phenotypes [151]. Likewise, experimental models of muscle degeneration have demonstrated that oxidative stress promotes mitochondrial dysfunction and myofiber damage [152,153] (Figure 6).

### 4.3. Potential Clinical Perspectives Focusing Therapeutic Strategies

Although, as mentioned before, current therapies focus on physiotherapy to manage symptoms, there is growing interest in targeted approaches based on oligonucleotides for disorders associated with dysregulation of RNA homeostasis (Figure 4). In this context, a study on myotonic dystrophy type 1 (DM1) highlighted the potential of novel strategies based on anti-microRNA (anti-miRs) molecules [154]. Anti-miRs are short RNA sequences designed to bind specific microRNAs (miRNAs), thereby relieving the repression of RNA-binding regulatory proteins targeted by these miRNAs [155] (Figure 5 and Figure 6).

Similarly, another study explored the use of short RNA chaperones to regulate TARBP/TDP-43 aggregates in the context of neurodegenerative diseases [156]. These short RNAs interact with and reinforce the RNA recognition motifs of TARBP/TDP-43. This interaction allosterically disrupts a conserved helical segment within the PRD and favors conformations that are less prone to aggregation [56]. Given that this strategy targets an RBP containing a PRD, applying the same proof of concept to the behavior of TIA1 [156] may yield promising results.

However, it should be noted that a major limitation of oligonucleotide-based therapies is their generally poor uptake by tissues, with skeletal muscle representing a particularly challenging target [156]. Notably, some studies have emphasized the use of lipid conjugation to enhance delivery and uptake in the muscle tissue [157]. Further, optimization of this kind of approach could provide a foundation for developing a potential therapeutic strategy for WDM.

Given that WDM is a muscle disorder, additional therapeutic strategies should focus on improving quality and function of mitochondria within muscle tissue. Altered mitochondrial morphology and function have been reported in WDM [157], as well as in several other muscle disorders [158,159,160]. While these strategies would not correct the primary molecular defect, they could still contribute to improving disease outcomes, given the essential involvement of mitochondria in processes such as autophagy, apoptosis, and cellular stress regulation [161,162].

Until recently, one of the main challenges associated with mitochondrial transplantation was the limited efficiency with which mitochondria were delivered to the target cells [163]. The development of the MitoCatch system has addressed part of this limitation by enhancing the therapeutic transfer of mitochondria, enabling their selective delivery to damaged cells, and promoting their functional integration within the recipient tissue [163] (Figure 5 and Figure 6).

Since changes in muscle strength can be subjective or slow to manifest during clinical examinations, imaging tools and overall quantitative muscle MRI (e.g., Dixon MRI techniques) are used to measure the exact muscle fat fraction. This allows researchers and clinicians to objectively track whether a trial drug or therapeutic management plan slows muscle degeneration over time.

### 4.4. Upcoming Milestones

To broaden the pathophysiological scope and address new challenges, several questions arise regarding critical mechanical, biochemical, and interactive aspects related to regulatory networks and crossroads, as well as specific vulnerabilities of the muscle fiber environment; in addition to the possible regulatory role linked to the expression of the TIA1 variant with the WDM mutation. We must bridge the gap between the ubiquitous nature of TIA1 and the tissue-specific (i.e., skeletal muscle) WDM phenotype. For example, addressing the “multisystemic issue” involves finding answers to the following questions: Why does a mutation in a ubiquitous protein, such as TIA1, selectively cause distal myopathy rather than a multisystemic disorder? Is this due to a specific mechanical stress in the skeletal muscle associated with the particular vulnerability of RNA metabolism in the distal muscle? Furthermore, is there tissue-specific expression of certain TIA1 isoforms or muscle-specific interaction partners of TIA1 or other RBPs that could explain the strict localization of the pathology to muscle fibers?

However, what do we know and not know about glucose metabolism and insulin signaling in patients with WDM? Currently, no published clinical studies, case series, or patient registries have specifically evaluated glucose metabolism, fasting insulin, OGTT, HbA1c, or insulin signaling pathway biomarkers in patients with WDM. Existing clinical literature on WDM has focused exclusively on neuromuscular phenotype. The harsh bibliographic reality is that there is a significant gap. Therefore, to date, neither historical nor current medical literature has described any cases of patients with WDM presenting with hyperglycemia, impaired insulin signaling, pancreatic endocrinopathies, or classic complications secondary to type 2 diabetes. WDM is clinically characterized by its progressive, late-onset, distal muscle phenotype (Figure 6).

These new and potential findings suggest that WDM is a multisystemic disorder involving possible interactions between various organ pathological systems, ranging from pancreatic dedifferentiation (i.e., pancreas and insulin signaling pathway) to progressive muscular dystrophy, along with the interplay of several regulatory pathways, such as insulin signaling, SMN2 gene splicing (i.e., SMN2 splicing-dependent pathway), and RNA granule-dependent stress responses (I.e., proteotoxicity pathway), including TIA1-associated SGs and P-bodies dynamics. This “multimodal hypothesis”, which poses a major challenge, has not yet been tested (Figure 6).

## Figures and Tables

**Table 1:** Representative subtypes of distal myopathies.

Age of Onset	Gene	Protein Function	Specific Distal Myopathy	Locus	Inheritance	Pathology	Initial Muscle Group Involved	CK	PHENOTYPE MIM Number/Cite
Childhood-adolescence	*MYH7*	Chemical energy into mechanical force	Laing	14q11.2	AD > AR	Nuclear internalization, fiber size variability, type 1 atrophy and rimmed vacuoles	Ankle dorsiflex or muscles, neck flexors	Normal/moderately elevated	608358/[17,19]
Early adulthood	*GNE*	Sialic acid synthesis, rate limiting enzyme sialylation of muscle glycans	Nonaka	9p13.3	AR	Fiber size variation, rimmed vacuoles, amyloid deposition	Ankle dorsiflex or and toe extensor weakness	Highly elevated	605820/[15,19]
	*DYSF*	Membrane repair	Miyoshi myopathy type 2/dysferlinopathy	2p13.2	AR	Fiber size variability, central nuclei, split fibers and infiltration processes	Plantar flexion weakness and calf muscle atrophy	Highly elevated	606768/[19,23]
Late adulthood	*TIA1*	Splicing regulation and translation repression	Welander	2p13.3	AD	Rimmed vacuoles, Type 1 atrophy, SG dynamics altered	Forearm and hand muscles, finger extensors	Normal/moderately elevated	604454/[9,19]
	*TTN*	Structure and flexibility of the sarcomere	Udd	2q31.2	AD	Fiber size variability, fiber atrophy	Ankle dorsiflex or muscles	Normal/moderately elevated	600334/[14,19,24]
	*LDB3*	Sarcomere structural integrity	Markesbery-Griggs	10q23.2	AD	Myofibrillar myopathy with protein accumulations, rimmed and non-rimmed vacuoles	Anterior compartment in legs	Normal/moderately elevated	609452/[19,25,26,27]

Abbreviation: *CK*, creatine kinase; *MYH7*, Myosin Heavy Chain 7; AD, Autosomal Dominant; AR, Autosomal recessive; *GNE*, Glucosamine (UDP-N-acetyl)-2-Epimerase/N-acetylmannosamine kinase.; *DYSF*, Dysferlin; *TIA1*, T-cell intracellular antigen 1; *TTN*, Titin; *LDB3*, LIM Domain Binding 3.

## Data Availability

No new data were created or analyzed in this study.
